# *De novo* transcriptome analysis and microsatellite marker development for population genetic study of a serious insect pest, *Rhopalosiphum padi* (L.) (Hemiptera: Aphididae)

**DOI:** 10.1371/journal.pone.0172513

**Published:** 2017-02-17

**Authors:** Xinle Duan, Kang Wang, Sha Su, Ruizheng Tian, Yuting Li, Maohua Chen

**Affiliations:** 1 State Key Laboratory of Crop Stress Biology for Arid Areas, Key Laboratory of Crop Pest Integrated Pest Management on the Loess Plateau of Ministry of Agriculture College of Plant Protection, Northwest A&F University, Yangling, China; 2 College of Bee Science, Fujian Agriculture and Forestry University, Fuzhou, China; USDA Agricultural Research Service, UNITED STATES

## Abstract

The bird cherry-oat aphid, *Rhopalosiphum padi* (L.), is one of the most abundant aphid pests of cereals and has a global distribution. Next-generation sequencing (NGS) is a rapid and efficient method for developing molecular markers. However, transcriptomic and genomic resources of *R*. *padi* have not been investigated. In this study, we used transcriptome information obtained by RNA-Seq to develop polymorphic microsatellites for investigating population genetics in this species. The transcriptome of *R*. *padi* was sequenced on an Illumina HiSeq 2000 platform. A total of 114.4 million raw reads with a GC content of 40.03% was generated. The raw reads were cleaned and assembled into 29,467 unigenes with an N50 length of 1,580 bp. Using several public databases, 82.47% of these unigenes were annotated. Of the annotated unigenes, 8,022 were assigned to COG pathways, 9,895 were assigned to GO pathways, and 14,586 were mapped to 257 KEGG pathways. A total of 7,936 potential microsatellites were identified in 5,564 unigenes, 60 of which were selected randomly and amplified using specific primer pairs. Fourteen loci were found to be polymorphic in the four *R*. *padi* populations. The transcriptomic data presented herein will facilitate gene discovery, gene analyses, and development of molecular markers for future studies of *R*. *padi* and other closely related aphid species.

## Introduction

The bird cherry-oat aphid, *Rhopalosiphum padi* (L.), is a notorious insect pest that devastates wheat crops globally [1–4]. This species can reduce both the yield and quality of wheat by sucking sap and transmitting barley yellow dwarf virus (BYDV), which leads to serious economic damage to wheat production [1]. In the past few years, due to global climate change, farming systems, wheat varieties, anthropogenic effects, and other factors, the damage to wheat caused by *R*. *padi* has increased and its distribution in China has expanded [5–8]. Due to its potential to cause serious damage to crops, the life cycle, host specificity, potential harmfulness, and methods of management of *R*. *padi* have been the subject of intensive studies [9–12]. Furthermore, the dual sexual and asexual reproductive mode of *R*. *padi* make this species an important model for the evolution of life-history traits and sympatric speciation [13–15].

Population genetic studies can provide insights into the evolution of reproduction modes, adaptive strategies of aphid species in agroecosystems, and the influence of environmental and anthropogenic factors on the genetic diversity and genetic structure of, as well as gene flows between, aphid populations [8]. These studies also facilitate the design and optimization of sustainable pest management strategies [8,13,16]. Molecular markers—such as restriction fragment length polymorphism (RFLP), random amplified polymorphic DNA (RAPD), mitochondrial DNA (mtDNA), and simple sequence repeats (SSRs)—are useful for studies of insect taxonomy, phylogeography, genetic diversity, and population structure at various taxonomic levels [16–18]. Microsatellite markers are special repetitive DNA sequences that have a high information content, co-dominance, locus specificity, and are readily amplified [19,20]. Therefore, microsatellite markers are used widely in meta-population genetics studies at various scales [3, 21–24]. Simon et al. (2001) isolated eight polymorphic microsatellite loci in *R*. *padi* and confirmed their utility for population genetics studies of parthenogenetic organisms [25]. Subsequently, these microsatellite loci were used in population genetics studies of French [25], Australian [17], and Chinese [8] *R*. *padi* populations. However, only five microsatellite loci were highly polymorphic in Chinese *R*. *padi* populations, which are insufficient for *R*. *padi* population genetics research [26].

Despite the abundance of microsatellite markers, traditional microsatellite development methods require expertise and substantial investment of time and other resources, particularly for non-model species lacking reference genomes and transcriptome data [27–30]. Next-generation sequencing (NGS) technologies are considered to be powerful, rapid, cost-effective, and reliable tools for transcriptional analysis, novel gene discovery, and molecular marker development [31–33]. Unlike traditional methods, transcriptome sequences generated by NGS facilitate rapid mining of microsatellite markers in non-model organisms [30,34]. Transcriptome sequences are coding DNA, and so a high proportion contains functional information [35,36]; therefore, transcriptome-derived microsatellite markers are situated close to or within functional genes [37–39] and increase cross-species transferability [40–42]. Transcriptomic sequencing for mining of microsatellite markers has been applied to several insect species, such as *Rhagoletis pomonella* [43], *Aphis glycines* [44], *Dolerus aeneus* [45], *Maruca vitrata* [46] and *Spodoptera exigua* [47].

In this study, the complete transcriptome of adult *R*. *padi* was characterized using the Illumina HiSeq 2000 platform, and all transcriptome sequences were assembled, BLAST searched, and annotated using public databases. Based on the transcriptome databases, several thousand *R*. *padi* microsatellite loci were mined, some of which were used to design primers to validate and estimate the intraspecific genetic diversity in four *R*. *padi* populations. To our knowledge, this is the first transcriptome analysis of this non-model species. The transcriptome data and polymorphic microsatellite markers will facilitate further studies of the population genetics and molecular biology of *R*. *padi*.

## Materials and methods

### Ethics statement

No specific permissions were required for the described field studies for this wide spread agriculture pest. We confirm that the locations were not privately owned or protected in any way. The field studies did not involve endangered or protected species.

### Insect materials, RNA extraction, cDNA library construction, and Illumina sequencing

A apterous parthenogenetic female of *R*. *padi* was collected from a wheat field at Northwest A&F University, Yangling, Shaanxi, China in July 2013 (108°05’E, 34°17’N) to set a clone (parthenogenetic line) on seedlings of wheat (*Triticum aestivum*) cultivar “Xiaoyan 22” at 24 ± 1°C, 40% RH, and a 16:8 h (L:D) photoperiod. Ten aphids were randomly selected from the clone for RNA extraction. The total RNA of *R*. *padi* was isolated using TRIzol reagent (Tiangen Biotech, Beijing, China) with minor modifications at the recovery step, in which RNase-free filter columns (Sangon Biotech, Shanghai, China) were used. RNA quantity and quality were assessed by gel electrophoresis and spectrophotometry, respectively. Ribosomal RNA (rRNA) was depleted from RNA samples using the Ribo-Zero™ rRNA Removal Kit (Human/Mouse/Rat) (Epicentre, Madison, USA) following the manufacturer's instructions. Around 20 μg purified RNA samples were sent to Beijing Genomics Institute (BGI) (Shenzhen, China) for cDNA library construction. Briefly, poly-T oligo-attached magnetic beads (Illumina, San Diego, CA, USA) were used to isolate poly (A) RNA from total RNA. Then a SuperScript Double-Stranded cDNA Synthesis kit (Invitrogen, Camarillo, CA) was employed for double-stranded cDNA synthesis with random hexamer (N6) primers (Illumina, San Diego, CA, USA). To normalize cDNA, the frequency of abundant cDNA species was reduced using Trimmer-2 cDNA Normalization Kit (Evrogen, Moscow, Russia). The T4 DNA polymerase, Klenow DNA polymerase and T4 polynucleotide kinase were used for end-repair and phosphorylation of synthesized cDNA. These repaired cDNA fragments were 3’ adenylated using Klenow Exo- (Illumina, San Diego, CA, USA). Illumina paired-end adapters were ligated to the ends of these 3’-adenylated cDNA fragments. The products of this ligation reaction were electrophoresed on a 2% (w/v) Tris-acetate-EDTA-agarose gel for downstream enrichment with different sizes. cDNA fragments of 200 (± 25 bp) were excised from the gel. Fifteen cycles of PCR amplification were performed with PCR primers (PE 1.0 and PE 2.0) and Phusion DNA Polymerase to enrich the quantity of purified cDNA template. The majority of the amplified fragments in the Illumina library were about 200 bp in size. Four samples were sequenced per lane on an Illumina HiSeq 2000 platform (Illumina Inc., San Diego, CA, USA). Paired-end sequencing was used to sequence both ends and the cDNA library was deep-sequenced generating four gigabytes of data and a total of 114,428,314 raw reads. After cleaning and trimming, a total 108,340,100 clean reads were used for assembly and analysis.

### *De novo* assembly and analysis of Illumina reads

To ensure the quality requirement for *de novo* transcriptome sequencing, a stringent filtering process was carried out. Initially, Illumina reads that passed the Failed-Chastity filter (Illumina) based on a setting of "failed-chastity less than or equal to 1" with a chastity threshold of 0.6, were reserved on the first 25 cycles. Then, all reads with adaptor contamination and low-quality reads with ambiguous sequences "N" were discarded. Finally, we ruled out reads with more than 10% Q < 20 bases. The cleaned reads were assembled de novo using SOAPdenovo2 and contigs with length less than 200 bp were discarded due to a low annotation rate [48]. The paired-end Illumina reads were first combined to produce longer fragments (*i*.*e*., contigs) and then mapped back to the contigs. The paired-end reads and contigs were assembled to form longer sequences that originated from the same transcript, with N indicating unknown bases (*i*.*e*., scaffolds). The paired-end reads were used for gap filling of the scaffolds to obtain unigenes with the least Ns that could not be extended at either end. All unigenes assembled were compared with the non-redundant protein database (nr) of the National Center for Biotechnology Information (NCBI), non-redundant nucleotide sequence (nt) database (NCBI) (http://www.ncbi.nlm.nih.gov), UniProt/Swiss-Prot (http://www.expasy.ch/sprot), and the Clusters of Orthologous Groups (COG) database (http://www.ncbi.nlm.nih.gov/COG/) using BLASTx [49] with E-values of less than 1e^–5^ and 1e^–10^, respectively. The COG functional classification was used to analyze the gene composition, as well as predict and classify possible functions of transcriptome sequences. With the nr annotation, Blast2GO was used for gene ontology (GO) annotation of the unigenes according to molecular function, biological process and cellular component ontologies (http://www.geneontology.org). The Kyoto Encyclopedia of Genes and Genomes (KEGG) pathway database and the online KEGG Automatic Annotation Server (http://www.genome.jp/tools/kaas/) were used to map unigenes to pathways applying an E-value threshold of 1e^–5^. During the determination of the sequence direction of unigenes, the priority order of nr, Swiss-Prot, KEGG and COG was followed when the alignment results of four databases conflicted with each other.

### SSR mining and primer design

The MIcroSAtellite (MISA, http://pgrc.ipk-gatersleben.de.sci-hub.org/misa/) Perl script was employed to identify microsatellites in the unigenes. In this study, cDNA-based SSRs (cSSRs) were considered to contain motifs with two to six nucleotides and a minimum of four contiguous repeat units. The criterion of no fewer than six repeat units for di-, five for tri- to tetra-, and four for penta- to hexa-nucleotide repeats was adopted. Based on the MISA results, repeat motifs were set randomly and searched in sequences longer than 200 bp. The frequency of cSSRs refers to kilobase pairs of cDNA sequences containing one SSR. The output was then parsed by the Primer3-2.3.4 program (http://sourceforge.net/projects/primer3/files/primer3/2.3.4/primer3-2.3.4.tar.gz/download) for design of PCR primers in the flanking regions of SSRs. Primers were designed based on the following criteria: (1) length of 18–23 bp; (2) annealing temperature of 55–65°C with a maximum discrepancy of <2°C among primers; and (3) a PCR product size of 80–300 bp. Finally, 60 pairs of primers were designed and screened for their PCR-amplification efficacy.

### PCR amplification and validation of selected SSRs

To evaluate the amplification efficacy, specificity, and polymorphisms of all selected SSRs, we collected *R*. *padi* samples from Lanzhou in the Gansu Province (the sample was coded as GSL; the coordinates are 103°41′ E and 36°05′ N), Xianyang in the Shaanxi Province (SAX; 108°05′ E, 34°17′ N); Wuhan in the Hubei Province (HUW; 112°47′ E, 32°08′ N), and Baicheng in the Jilin Province (JLB; 122°52′ E, 45°39′ N). Apterous parthenogenetic individuals from the four regions were brought to the laboratory in individual tubes. Twelve individuals from each region were used to set up 12 respective clones (parthenogenetic lines) on seedlings of wheat cultivar ‘Xiaoyan 22’ at 24±1°C, 40% RH, and a 16:8 h (L: D) photoperiod. A total of 48 clones were set up for the four regions. One individual was randomly taken from the first generation of each clone for DNA extraction. Genomic DNA was extracted from each individual using the EasyPure Genomic DNA Kit (TransGen Biotech, BeijingChina) according to the bench protocol for animal tissues. DNA was eluted in 30 μL of ultrapure water and stored at –20°C. Three primers-a forward primer with an M13 (-21) at the 5’ end, a reverse primer, and an FAM fluorscent dye-labeled M13 (-21) primer [50] were used for PCR amplification of the microsatellite loci. PCR was performed in a total volume of 25 μL, comprising 12.5 μL of 2× Taq Master Mix (containing 0.05 U/μL Taq DNA Polymerase, 2× Taq PCR Buffer, 3 mM MgCl_2_, and 400 μM dNTP mix) (CoWin Biotech, Beijing, China), 0.5 μL of each forward primer (10 μM), 2 μL of each reverse primer (10 μM), 2 μL of M13 primer (10 μM), and 1.5 μL of genomic DNA (10–30 ng/μL). PCR amplification involved denaturation at 95°C for 2 min, followed by 30 amplification cycles consisting of 95°C for 20 s, 20 s at the primer-specific annealing temperature (Table 1), and 72°C for 20 s. This was followed by eight cycles of 95°C for 30 s, 53°C for 45 s, and 72°C for 45 s, and a final step at 72°C for 10 min. To examine the length of the amplified PCR products, an ABI3730XL automated DNA sequencer (Applied Biosystems, Foster City, CA, USA) was used, and all genotypes were called with GeneMapper v4.0 software (Applied Biosystems, Foster City, CA, USA).

**Table 1:** Characteristics of 24 microsatellite loci developed for *R*. *padi*.

Locus	Primer sequence (5'-3')	Repeat motif	Ta (°C)	Size range (bp)
RP02	F: TACACACTTTGCCGTAAATCAGA; R: CGTTGTATTGGCATGATATAGG	(AC)_8_	56	218–234
RP05	F: GCGGTCTTTCGTTTCTCTCTATC; R: GGGAAATTGAAATAACATCTCGG	(TC)_11_	60	145–167
RP06	F: ATAAAGGTACCTACGCGAAATCC; R: CCTCGTGACTCGACATGATAGTA	(GT)_10_	60	148–168
RP08	F: TCATTTGCGTATAAGACATGGAT; R: CATCACTGCATATCAGTCTGACG	(AC)_10_	54	133–153
RP11	F: GGGTCGGGTATAGTCAGAGTCTT; R: CTCGACGACAATTCTACGTCTTT	(GT)_10_	56	138–158
RP13	F: AGTTGTATTGTTTTGAACGGTCG; R: TCGTGGATTATCGTTACAATACTGA	(AT)_11_	58	146–166
RP14	F: TTGTGAAATCGGTTTTACGTTTT; R: CTCTACACTCAAGCCCCAATTTA	(GT)_10_	56	146–166
RP17	F: AGTAAGTCCGTCCCGTCGTCT; R: AATATCGCGTATCGTACCAGTGT	(CAG)_7_	58	140–170
RP22	F: TTCGATCTGTTTCTTGAGCG; R: CGCGGTATAAGGTCACCG	(GCC)_8_	56	147–171
RP23	F: CTGAGCTGCAGTATTTTCCGAGT; R: CGCGTGCATAAATGTATAACGTA	(ATAC)_5_	54	150–170
RP24	F: AGTTGGGGAATTTATAGTGAGCG; R: TGGTGTCTCAGAGTAAAGAAAAGAA	(TATG)_5_	58	146–166
RP25	F: ATTTTGAGTTTTCACCATCGTTT; R: TTTTTAGTACGCCAAATTGTTGA	(GGTA)_5_	58	145–165
RP29	F: TTAAATAAAAAGGCCAAACCCAT; R: ATTGGCGCTAAATTAAATGACTC	(ATCAGA)_4_	56	146–170
RP30	F: AACGACCCAGATTATGTAGTCCA; R: ACCAACACCAACACCAACATC	(TGGTGT)_4_	56	144–168
RP31	F: AGCAGCTTCTTGAGCTGGAC; R: AACAACAACACCAAAGGGTCTAC	(GCC)_8_	56	143–167
RP36	F: AATCATACAAACGAGATTCTTCCC; R: GCAAGTATCGAAACGCTAACCTA	(TA)_6_	56	224–236
RP37	F: AGTGTAAATGATATCGCGGCTTA; R: TCCGGTTCACAGTACAAAAATATC	(AT)_8_	56	210–226
RP38	F: ATACATAGGACACAGGCGAAAAC; R: CACCACCTAAATTGTATTAAGGAAA	(AT)_7_	56	203–217
RP42	F: GATCACAATAATATTTACCGGCG; R: ATAACGTACGCGTGTGGATAAAA	(TA)_10_	54	129–149
RP43	F: GTATGCGGTCAACCTATTTTACG; R: TATTGTTGGAATTAGAAAGGCCA	(AT)_10_	56	120–140
RP45	F: ACCATGATCAACTTAAGAGGTGC; R: ATGTTTACGTTTAGAGCGCATGT	(TA)_10_	60	125–145
RP47	F: TATTTACGACGGGAATGTACAGC; R: TCAATATATCTCTCACCCCCTCA	(TATC)_5_	60	143–163
RP48	F: GTTCCTCCGGCCGTACAT; R: GATATCTCGTTCTGGCGAGTCT	(CAC)_8_	58	141–165
RP60	F: ATTTAGATTTCATCTCATCGTCGG; R: GCTGAGGACGAAAACTATTTTGA	(CGT)_7_	56	145–172

### Statistical analysis

Population genetic parameters for polymorphic loci, such as the observed heterozygosity (Ho), the expected heterozygosity (He), and number of alleles (Na) were calculated using FSTAT v2.9.3.2 software [51]. The Excel Microsatellite Toolkit v3.1 program (MS Tools) [52] was utilized to calculate the polymorphism information content (PIC) of each SSR; the inbreeding index (Fis) was assessed using Popgene v1.3.1 software [53]; Hardy-Weinberg equilibrium (HWE) and *P* values of HWE were evaluated using GENEPOP v4.0 software [54]. The statistical power of microsatellite markers for detecting genetic differentiation at various Fst (fixation index) levels was evaluated using POWSIM v4.1 software [55]. Based on an effective population size of Ne = 1000, simulations were carried out with Fst values of 0.0001, 0.0025, 0.005, 0.0075, 0.01, 0.0125, 0.015, 0.0175, and 0.02, and 1000 replicates [55]. Chi-squared tests and Fisher’s exact tests were used to analyze the allele number and frequency at randomly selected microsatellite loci, and the power of the analysis was indicated by the proportion of tests that were significant at *P* < 0.05 [8].

## Results

### Illumina sequencing and de novo assembly of short reads

**Fig 1:**
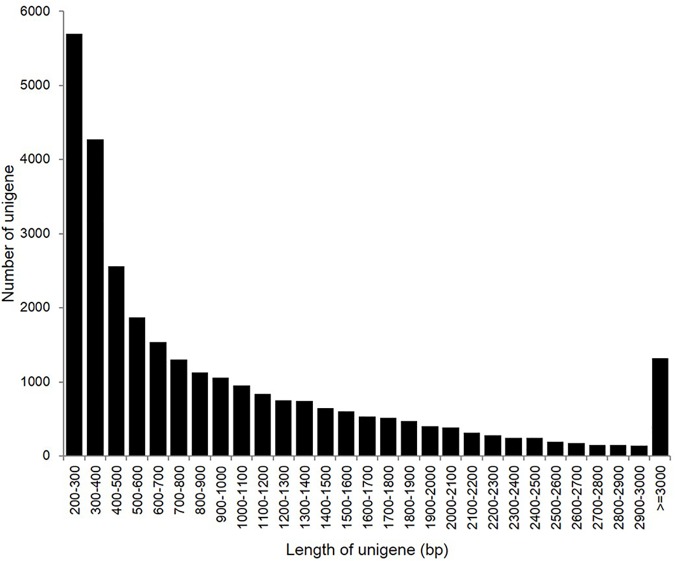
Frequencies length distribution of Illumina read sequences.

Illumina sequencing resulted in 114,428,314 raw reads of which 108,340,100 were of acceptable quality. Total GC content was 40.03%, with a Q20 value of 98.37% and a final length of 9,750,609,000 bp (S1 Table). The transcriptomic data are available via NCBI Short Read Archive (SRA) with accession numbers of SPR095727, SPR5133458 and SPR5133459. The average raw read length was 90 bp, which is consistent with the Illumina sequencing capacity. The raw reads were assembled into contigs using the SOAPdenovo2 software, and empty reads and low-quality sequences were filtered out. These contigs were further assembled into scaffolds by paired-end joining and gap-filling. Finally, the scaffold sequences were assembled into clusters, which yielded 29,467 unigenes with an average length of 990 bp (N50 = 1,580) and main lengths between 200-3000bp. Of these unigenes, 5,699 (19.34%) were 200–300 bp, 6,832 (23.19%) were 301–500 bp, 6986 (23.4%) were 501–1,000 bp, 6448 (21.89%) were 1,001–2,000 bp, 2273 (7.71%) were 2,001–3,000 bp, and 1319 (4.48%) were >3,000 bp in length (Fig 1).

### Functional annotation of the unigenes

All *R*. *padi* unigenes were aligned to the nr, nt, UniProt, KEGG, COG, and GO databases to predict and classify possible functions. A total of 24,302 (82.47%) unigenes were annotated by BLAST searches of the databases. Among these unigenes, 21,139 (71.74%), 22,939 (77.85%), 16,024 (54.38%), 14,586 (49.5%), 8,022 (27.22%), and 9,895 (33.58%) had homologous sequences in the nr, nt, Swiss-Prot, KEGG, COG, and GO databases, respectively. In addition, 5,165 (17.53%) unigenes showed no homology to known sequences.

**Fig 2:**
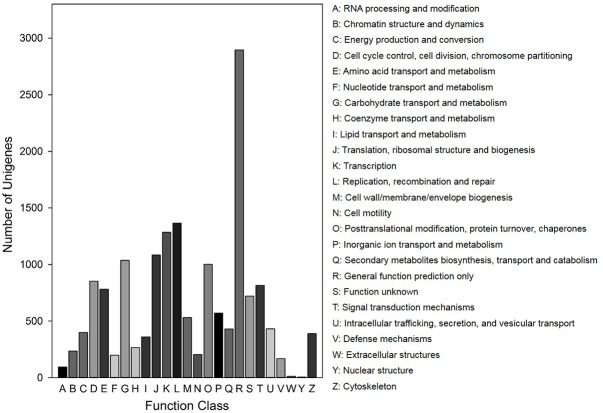
Histogram presentation of clusters of orthologous groups (COG) classification.

In the COG functional classification, 8,022 generated 91,152 functional annotations across 25 COG categories (Fig 2). Among these COG categories, general function prediction contained the largest number of unigenes (2,896, 36.10%), followed by replication, recombination, and repair (1,365, 17.02%), transcription (1,285, 16.02%), translation, ribosomal structure, and biogenesis (1,083, 13.50%), and other groups. Interestingly, only six and 14 unigenes were related to nuclear structures and extracellular structures, respectively.

**Fig 3:**
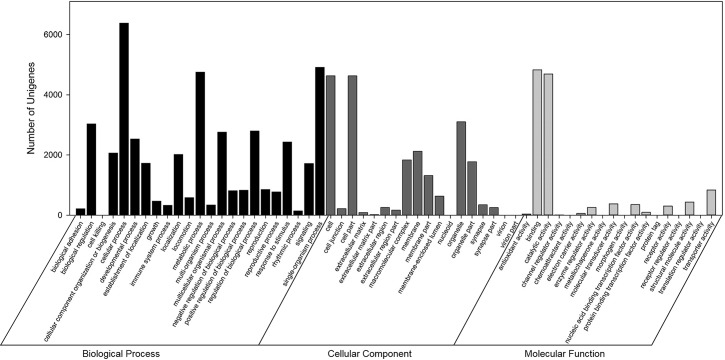
Gene Ontology classification of assembled unigenes.

A total of 9,895 unigenes with BLAST matches to known proteins were annotated with 76,184 GO functions based on sequence similarity, with an average of 7.7 GO annotations per unigene (Fig 3). The three main GO annotation categories were biological process (42,447, 55.72%), cellular component (21,425, 28.12%), and molecular function (12,312, 16.16%). The annotations could be further subdivided into 59 subcategories (Fig 3). For sequences that initially sorted to the biological process classification, cellular process, single-organism process, metabolic process, and biological regulation were among the most represented matches. The major subcategories for the cellular component classification were cell and cell part, whereas in the molecular function classification the major subcategories were binding and catalytic activity.

In addition, 14,586 unigenes aligned with 257 KEGG pathways (S2 Table). These pathways contained 1–2,105 unigenes. The pathways with the greatest number of unigenes were metabolic pathways (2,105 unigenes, 14.43%), RNA transport (505, 3.46%), regulation of actin cytoskeleton (474, 3.25%), focal adhesion (466, 3.19%), and pathways in cancer (461, 3.16%). Only one unigene was involved in each of the D-glutamine and D-glutamate metabolism, allograft rejection, and graft-*versus*-host disease pathways.

### Development and characterization of microsatellite markers

To develop novel molecular markers, all of the 29,476 unigenes were used to mine potential microsatellite loci consisting of di- to hexa-nucleotide SSRs with at least four repetitions. Using the MISA Perl script, a total of 7,936 potential SSRs were identified in 5,564 unigenes, 3,960 contained only one SSR while 1,604 contained more than one SSR. Of these SSRs, 714 were present in compound form (with adjacent tandem simple repeats of a different sequence), and 7,222 SSRs were in perfect form (without interruptions in the runs of repeats) [56]. In addition, the frequency, type, and distribution of the potential 7,936 SSRs were analyzed. On average, one SSR was present every 0.99 kb in unigenes, and the frequency of cSSRs was 26.93%.

**Fig 4:**
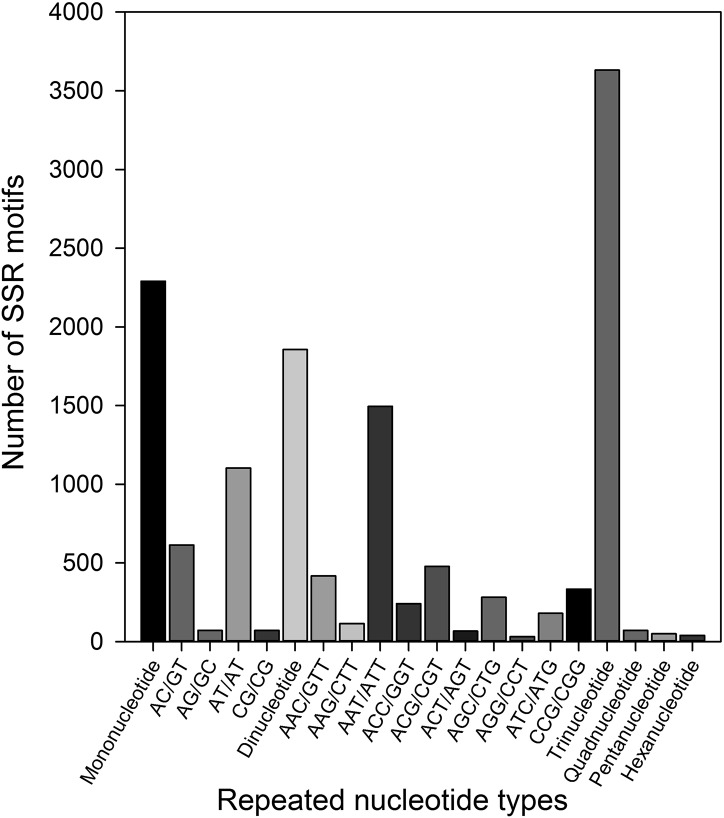
Total numbers of SSRs based on motif types in *R*. *padi*.

Among the 7,936 SSRs, the tri- and mono-nucleotide repeat motifs were most abundant (3,631, 45.75% and 2290, 28.86%, respectively), followed by di- (1,857, 23.40%), tetra- (70, 0.88%), penta- (49, 0.62%), and hexa-nucleotide (39, 0.49%) repeat motifs (Fig 4). Di- to hexa-nucleotide motifs were further analyzed in terms of the number of repeat units (or SSR length, Table 2). The most frequent number category of repeat units was 4–9, accounting for 68.18% of total SSRs, followed by the 10–15 repeat unit category (1793 SSRs, 22.59%). There were 732 SSRs with more than 15 repeat units. Among the detected SSRs, 163 motif types were identified, that comprised 4, 12, 59, 32, 28, and 28 types of mono- di-, tri-, tetra-, penta-, and hexa-nucleotide repeats, respectively. The A/T mono-nucleotide repeat was the most abundant motif type (2,267, 28.57%), followed by AAT/ATT (1,494, 18.83%), AT/TA (1,103, 13.9%), AC/GT (612, 7.71%), ACG/CGT (447, 6.01%), AAC/GTT (417, 5.25%), CCG/CGG (332, 4.18%), AGG/CTG (281, 3.54%), and ACC/GGT (240, 3.02%). The remaining 154 motif types accounted for 8.98% of the total (Fig 4).

**Table 2:** Frequency of SSRs based on repeat types in *R*. *padi* transcriptome.

Repeat type	No. of repeat														
	4	5	6	7	8	9	10	11	12	13	14	15	>15	Total	Percentage
Mononucleotide	0	0	0	0	0	0	0	0	696	413	262	187	732	2290	28.86%
Dinucleotide	0	0	715	441	263	203	121	105	9	0	0	0	0	1857	23.40%
Trinucleotide	0	1876	1039	608	108	0	0	0	0	0	0	0	0	3631	45.75%
Quadnucleotide	0	62	8	0	0	0	0	0	0	0	0	0	0	70	0.88%
Pentanucleotide	43	6	0	0	0	0	0	0	0	0	0	0	0	49	0.62%
Hexanucleotide	39	0	0	0	0	0	0	0	0	0	0	0	0	39	0.49%
Total	82	1944	1762	1049	371	203	121	105	705	413	262	187	732	7936	
Percentage	1.03%	24.50%	22.20%	13.22%	4.67%	2.56%	1.52%	1.32%	8.88%	5.20%	3.30%	2.36%	9.22%		

### Validation of microsatellite markers

**Fig 5:**
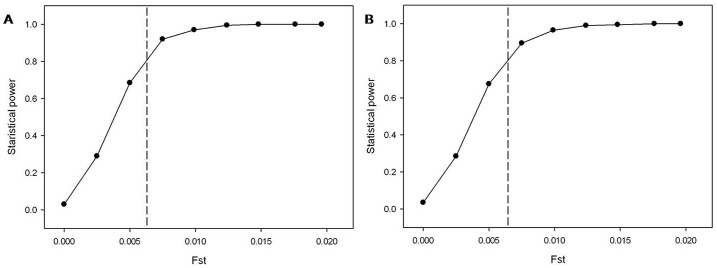
POWSIM analyses for testing power to detect genetic differentiation at different Fst values for 14 microsatellite loci as showed by Chi-square test (A) and Fisher’s exact test (B).

A total of 60 microsatellite loci were selected at random for verification of their utility in various *R*. *padi* populations. Twenty-four of these loci were amplified successfully and yielded bands of the correct sizes; these loci were used to analyze the genetic diversity of *R*. *padi* (Table 1). Fourteen loci (RP06, RP08, RP13, RP14, RP22, RP23, RP24, RP30, RP31, RP42, RP43, RP45, RP48 and RP60) were polymorphic in four different geographical populations of *R*. *padi* (Table 3). The number of alleles (Na) per locus ranged from 2 to 9, with an average of 3.98. The observed heterozygosity (Ho) value was 0.417–1.000, whereas the expected heterozygosity (He) value was 0.409–0.823. The the polymorphism information content (PIC) of each locus ranged from 0.221 to 0.765. The inbreeding index (Fis) values ranged from –1.000 to 0.367, most of which were negative values indicating the heterozygous excess in the four *R*. *padi* populations. Among the 14 analyzed microsatellite loci in the four *R*. *padi* populations, 10 showed deviation from Hardy-Weinberg equilibrium (HWE) (*P* < 0.005) (Table 3). Power calculations using a chi-squared test (Fig 5A) and Fisher’s exact test (Fig 5B) showed that Fst (fixation index) values as low as 0.0062 could be detected with more than 80% probability. Therefore, the 14 microsatellite loci provided sufficient statistical power to detect population differentiation.

**Table 3:** Genetic diversity revealed by 14 microsatellites in four populations of *R*. *pad*i from China.

Locus	P 1					P 2					P 3					P 4				
	Na	Ho	He	PIC	Fis	Na	Ho	He	PIC	Fis	Na	Ho	He	PIC	Fis	Na	Ho	He	PIC	Fis
RP06 (KU325537)	7	0.750	0.725	0.665	-0.037	6	0.583	0.801	0.730	0.280***	5	0.583	0.565	0.488	-0.034	5	1.000	0.670	0.575	-0.526**
RP08 (KU325538)	3	0.833	0.554	0.428	-0.538	2	0.917	0.518	0.373	-0.833*	2	0.917	0.518	0.373	-0.833*	3	0.667	0.554	0.428	-0.214
RP13 (KU325539)	4	1.000	0.652	0.559	-0.571*	5	0.833	0.634	0.571	-0.333*	3	1.000	0.594	0.477	-0.737**	4	0.917	0.656	0.579	-0.424
RP14 (KU325540)	4	0.667	0.540	0.482	-0.248	3	0.250	0.359	0.307	0.313*	3	0.750	0.562	0.480	-0.356	2	0.583	0.431	0.328	-0.375
RP22 (KU325541)	4	0.833	0.612	0.535	-0.384	5	0.583	0.819	0.750	0.297**	4	0.636	0.680	0.594	0.067	5	0.455	0.706	0.620	0.367*
RP23 (KU325542)	5	0.833	0.710	0.643	-0.183	6	0.917	0.725	0.644	-0.280***	4	0.833	0.717	0.638	-0.170*	5	0.667	0.580	0.513	-0.158
RP24 (KU325543)	2	0.750	0.489	0.359	-0.571	3	0.750	0.518	0.408	-0.478	2	1.000	0.522	0.375	-1.000**	2	0.417	0.344	0.275	-0.222
RP30 (KU325544)	3	0.750	0.554	0.468	-0.375	2	0.667	0.464	0.346	-0.467	3	0.500	0.409	0.341	-0.234	3	0.667	0.522	0.449	-0.294
RP31 (KU325545)	5	1.000	0.699	0.627	-0.459	4	0.917	0.714	0.633	-0.301*	4	0.917	0.685	0.595	-0.360	5	0.583	0.623	0.553	0.067
RP42 (KU325546)	7	0.909	0.823	0.755	-0.111	9	1.000	0.823	0.765	-0.228	6	0.833	0.710	0.643	-0.183	4	0.833	0.641	0.557	-0.317
RP43 (KU325547)	3	0.500	0.409	0.341	-0.234	4	0.583	0.486	0.432	-0.213	3	0.500	0.409	0.341	-0.234	4	0.250	0.239	0.221	-0.048
RP45 (KU325548)	5	1.000	0.732	0.659	-0.389	6	1.000	0.696	0.612	-0.467**	3	0.917	0.583	0.470	-0.613*	6	0.546	0.537	0.491	-0.017
RP48 (KU325549)	3	0.833	0.540	0.420	-0.583	3	0.417	0.467	0.397	0.113	3	0.583	0.475	0.410	-0.242	4	0.667	0.533	0.469	-0.266
RP60 (KU325550)	2	1.000	0.524	0.375	-1.000**	4	0.667	0.511	0.431	-0.323	4	0.833	0.605	0.523	-0.401	3	0.750	0.518	0.408	-0.478

Na, numbers of alleles; Ho, observed heterozygosity; He, expected heterozygosity; PIC, polymorphism information content; Fis, the inbreeding index; Deviation from Hardy-Weinberg equilibrium (HWE) are indicated by asterisks (*, *P* < 0.05, **,*P* < 0.01, ***, *P* < 0.001). GenBank accession number for each locus was showed in the parenthesis. The population P 1 was from Gansu Province, P 2 was from Shaanxi Province, P 3 was from Hubei Province, and P 4 was from Jiilin Province. Twelve individuals from each populations were used in the test. Detailed information of the sampling locations was described in the text.

## Discussion

### Transcriptome analysis

In this study, the complete *R*. *padi* transcriptome was sequenced on the Illumina HiSeq 2000 platform, yielding a total of 9,750,609,000 bp with 114.43 million clean reads. These sequences also produced longer unigenes (mean = 990 bp) than those assembled in *Ipomoea batatas* (765 bp) [57], *Eucalyptus grandis* (197 bp) [58], *Acropora millepora* (440 bp) [59], *Bemisia tabaci* (266 bp) [60], and *Dialeurodes citri* (539 bp) [61]. Furthermore, the N50 length of the unigenes was 1,580 bp, longer than that of *Ipomoea batatas* (765 bp) [57], *D*. *citri* (632 bp) [61], and *Grapevine phylloxera* (936 bp) [62]. This result indicated that the *R*. *padi* transcriptome sequences were of high quality, which was likely due to the improved transcript construction and scaffolding and low heterozygosity of the new paired-end sequencing technology.

For functional annotation, we utilized several complementary approaches to annotate the assembled sequences using several public databases. About 82.47% of the unigenes had orthologs or homologs in these databases and were assigned at least one functional annotation, which is higher than previous reports of other species using the same sequencing platform [39,60,61,63]. The high level of annotations is due to the availability of complete functional information in all of the public databases, the high mean length of unigenes [39], and the availability of an aphid genome database (http://www.aphidbase.com/aphidbase/). A total of 17.53% unigenes were unmapped in any of the databases, possibly due to the short sequence reads generated [61], the presence of non-coding transcripts among the unigenes [31], and/or the incompleteness of the public sequence databases [30]. In COG and GO functional classification, a large proportion of unigenes (27.22 and 33.58%) were assigned to a wide range of COG and GO classifications (Figs 2 and 3), indicating that the transcriptome data included a wide diversity of transcripts. In the KEGG pathway analysis, a high proportion of unigenes were mapped to metabolic pathways, the RNA transport pathway, and regulation of the actin cytoskeleton pathway (S2 Table). In addition, several pathways related to pesticide resistance—such as ABC transporters, drug metabolism-cytochrome P450, and metabolism of xenobiotics by cytochrome P450—were identified. These annotated unigenes will facilitate more in-depth investigations of population genetics and functional genomics of *R*. *padi* and other closely related aphid species.

### Microsatellite loci characterization

The major traditional methods for microsatellite loci development are the hybrid capture method [64], loci selection from available genetic/genomic information [65], and loci transferable from closely related species [28,66]. Compared with traditional methods, *de novo* transcriptome sequencing technology is a rapid, cost-effective, and reliable tool that enables microsatellite markers to be developed directly from transcriptome sequences, particularly for non-model species [33,42,67]. Among the 29,467 assembled unigenes, 5,564 (18.89%) possessed 7,936 potential microsatellite loci. This is higher than the values for other insect pests, such as *Bombyx mori* [68], *Tomicus yunnanensi* [69], *Bactrocera dorsalis* [70], and *Phenacoccus solenopsis* [71]. Six types of microsatellite loci repeat type were identified among the unigenes; the most common were trinucleotide (45.75%) and mononucleotide (28.86%) repeats, in agreement with the results for *D*. *aeneus* [45], *Timema cristinae* [72], and *B*. *dorsalis* [70]. Wang et al. (2012) found that trinucleotide microsatellite loci were abundant in the transcriptome data of *Tetrao tetrix*, and predicted that tri-nucleotides can remain in coding regions without causing reading frame shifts [73]. Therefore, the abundance and frequency of the various microsatellite loci repeat types were related to the size of the transcriptome database, the microsatellite loci search software used, and the parameter criteria [74]. A/T, AAT/ATT, and AT/TA were the most abundant SSR motif types in the *R*. *padi* transcriptome database. A/T homopolymers are also more abundant than C/G homopolymers in *Schistosoma mansoni* [75], *Tenebrio molitor* [76], and *P*. *solenopsis* [71]. Tóth et al. (2000) examined the abundance of microsatellites with repeated unit lengths of 1–6 base pairs in several eukaryotic taxonomic groups [77]. They found poly (A/T) tracts are more abundant in each taxon than poly(C/G) sequences and the plausible explanation for the higher proportion of A/T-rich SSRs is the poly-A tails of retroposed sequences and processed pseudogenes. In 154 non-model eukaryote species, the previous reports found that the GC/CG motif was rare, and that the GC/CG was absent in several eukaryote species [78–83]. Indeed, the CG/CG motif was detected at a low frequency (0.89%) in this study. This phenomenon cannot be explained only by the low C/G content of the genome and thus may represent a genuine pattern [78,84,85].

### Microsatellite loci development and validation

Among 60 randomly selected potential microsatellite markers, 24 loci (40%) were amplified successfully, and 14 loci exhibited polymorphisms in four *R*. *padi* populations. The low amplification rate may be caused by the special structure at the primer (s) location or between the primers, for example, the presence of large intron between primers, or unrecognized splice sites disrupting primer positions. The chimeric primers and assembly errors also could result in failed amplification. However, the other microsatellites obtained from our transcriptome data can provide a larger pool for mining more polymorphic loci. SSR polymorphisms are positively correlated with the number of motif repeats [70]. In the transcriptome database, the number of motif repeats of most SSRs (68.18%) ranged from 4 to 9, and only 9.23% of SSRs had more than 15 repeats. Hence, SSRs in the transcriptome were less polymorphic than genomic SSRs, but possessed potential polymorphisms [30, 86]. Among the four *R*. *padi* populations, 14 loci had fewer alleles (Na) than genomic SSRs, but similar Ho, He, and Fis values [8]. The PIC is an important parameter for microsatellite polymorphisms [87]. The average PIC values of 14 loci in four *R*. *padi* populations were 0.334–0.680, suggesting that 14 were moderately or highly polymorphic. A chi-squared test and Fisher’s exact test confirmed that 14 loci had strong statistical power to detect low Fst levels, while amplification at various annealing temperatures using the remaining 36 primer pairs failed.

## Conclusions

To our knowledge, this is the first report of the assembly and characterization of the transcriptome of *R*. *padi* using the Illumina HiSeq 2000 platform. A total of 2,9467 unigenes were generated and 7,936 EST-SSRs were identified, which will facilitate development of molecular markers for *R*. *padi*. Sixty of these loci were selected randomly, and 24 were amplified successfully and validated experimentally in four *R*. *padi* populations. Our results will enable development of microsatellite markers and population genetic studies of *R*. *padi*.

## Supporting information

S1 Table Summary of transcriptome data for *R*. *padi* and bioinformatics annotation. (PDF)

S2 Table KEGG biochemical mappings for *R*. *padi*. (PDF)
