# Supplementary material for: De novo transcriptome analysis and microsatellite marker development for population genetic study of a serious insect pest, Rhopalosiphum padi (L.) (Hemiptera: Aphididae)
Source: PLoS One. 2017 Feb 17;12(2):e0172513. doi: 10.1371/journal.pone.0172513 (PMC5315398; doi:10.1371/journal.pone.0172513)
Supplement: S1 Table — (PDF) [file pone.0172513.s001.pdf]

S1 Table. Summary of transcriptome data for *R. padi* and bioinformatics annotation

Data generation and filtering	
Raw sequences and assembly statistics	
Total Raw Reads	114,428,314
Total Clean Reads (length)	108,340,100 (9,750,609,000 bp)
GC percentage	40.04%
Q20 percentage	98.37%
N percentage	0.00%
Contigs(mean length; N50)	53,608 (423 bp, 939)
Unigenes (mean length; N50)	29,467 (990 bp, 1,580)
Bioinformatics annotations of unigenes	
All annotated unigenes	24,302 (82.47%)
Gene annotation against animal proteins of nr	21,139 (71.74%)
Gene annotation against animal protein of nt	22,939 (77.85%)
Gene annotation against Swiss-Prot	16,024 (54.38%)
Gene annotation against KEGG	14,586 (49.5%)
Gene annotation against COG	8,022 (27.22%)
Gene annotation against GO	9,895 (33.58%)
