# Supplementary material for: De novo transcriptome analysis and microsatellite marker development for population genetic study of a serious insect pest, Rhopalosiphum padi (L.) (Hemiptera: Aphididae)
Source: PLoS One. 2017 Feb 17;12(2):e0172513. doi: 10.1371/journal.pone.0172513 (PMC5315398; doi:10.1371/journal.pone.0172513)
Supplement: S2 Table — (PDF) [file pone.0172513.s002.pdf]

S2 Table KEGG biochemical mappings for *R. padi*

Pathway	Unigene number	Pathway ID	Level 1	Level 2
Regulation of actin cytoskeleton	474	ko04810	Cellular Processes	Cell motility
Focal adhesion	466	ko04510	Cellular Processes	Cell communication
Tight junction	343	ko04530	Cellular Processes	Cell communication
Endocytosis	337	ko04144	Cellular Processes	Transport and catabolism
Lysosome	274	ko04142	Cellular Processes	Transport and catabolism
Phagosome	228	ko04145	Cellular Processes	Transport and catabolism
Cell cycle	217	ko04110	Cellular Processes	Cell growth and death
Adherens junction	205	ko04520	Cellular Processes	Cell communication
Oocyte meiosis	195	ko04114	Cellular Processes	Cell growth and death
Peroxisome	161	ko04146	Cellular Processes	Transport and catabolism
Gap junction	117	ko04540	Cellular Processes	Cell communication
p53 signaling pathway	97	ko04115	Cellular Processes	Cell growth and death
Apoptosis	64	ko04210	Cellular Processes	Cell growth and death
Regulation of autophagy	30	ko04140	Cellular Processes	Transport and catabolism
MAPK signaling pathway	336	ko04010	Environmental Information Processing	Signal transduction
Calcium signaling pathway	286	ko04020	Environmental Information Processing	Signal transduction
Wnt signaling pathway	237	ko04310	Environmental Information Processing	Signal transduction
Neuroactive ligand-receptor interaction	218	ko04080	Environmental Information Processing	Signaling molecules and interaction
ECM-receptor interaction	179	ko04512	Environmental Information Processing	Signaling molecules and interaction
ABC transporters	175	ko02010	Environmental Information Processing	Membrane transport
Phosphatidylinositol signaling system	149	ko04070	Environmental Information Processing	Signal transduction
ErbB signaling pathway	124	ko04012	Environmental Information Processing	Signal transduction
TGF-beta signaling pathway	116	ko04350	Environmental Information Processing	Signal transduction
VEGF signaling pathway	102	ko04370	Environmental Information Processing	Signal transduction
Cell adhesion molecules (CAMs)	100	ko04514	Environmental Information Processing	Signaling molecules and interaction
NF-kappa B signaling pathway	99	ko04064	Environmental Information Processing	Signal transduction
Notch signaling pathway	96	ko04330	Environmental Information Processing	Signal transduction
Jak-STAT signaling pathway	95	ko04630	Environmental Information Processing	Signal transduction
Hedgehog signaling pathway	82	ko04340	Environmental Information Processing	Signal transduction
mTOR signaling pathway	76	ko04150	Environmental Information Processing	Signal transduction
Cytokine-cytokine receptor interaction	71	ko04060	Environmental Information Processing	Signaling molecules and interaction
MAPK signaling pathway - fly	51	ko04013	Environmental Information Processing	Signal transduction
RNA transport	505	ko03013	Genetic Information Processing	Translation
Spliceosome	391	ko03040	Genetic Information Processing	Transcription

Protein processing in endoplasmic reticulum	319	ko04141	Genetic Information Processing	Folding, sorting and degradation
mRNA surveillance pathway	285	ko03015	Genetic Information Processing	Translation
Ubiquitin mediated proteolysis	279	ko04120	Genetic Information Processing	Folding, sorting and degradation
Ribosome biogenesis in eukaryotes	215	ko03008	Genetic Information Processing	Translation
RNA degradation	183	ko03018	Genetic Information Processing	Folding, sorting and degradation
Ribosome	175	ko03010	Genetic Information Processing	Translation
RNA polymerase	145	ko03020	Genetic Information Processing	Transcription
Aminoacyl-tRNA biosynthesis	130	ko00970	Genetic Information Processing	Translation
Fanconi anemia pathway	125	ko03460	Genetic Information Processing	Replication and repair
Basal transcription factors	94	ko03022	Genetic Information Processing	Transcription
Nucleotide excision repair	86	ko03420	Genetic Information Processing	Replication and repair
DNA replication	85	ko03030	Genetic Information Processing	Replication and repair
Base excision repair	75	ko03410	Genetic Information Processing	Replication and repair
Homologous recombination	74	ko03440	Genetic Information Processing	Replication and repair
SNARE interactions in vesicular transport	63	ko04130	Genetic Information Processing	Folding, sorting and degradation
Mismatch repair	51	ko03430	Genetic Information Processing	Replication and repair
Proteasome	45	ko03050	Genetic Information Processing	Folding, sorting and degradation
Protein export	37	ko03060	Genetic Information Processing	Folding, sorting and degradation
Non-homologous end-joining	28	ko03450	Genetic Information Processing	Replication and repair
Sulfur relay system	18	ko04122	Genetic Information Processing	Folding, sorting and degradation
Pathways in cancer	461	ko05200	Human Diseases	Cancers: Overview
HTLV-I infection	392	ko05166	Human Diseases	Infectious diseases: Viral
Huntington's disease	361	ko05016	Human Diseases	Neurodegenerative diseases
Epstein-Barr virus infection	357	ko05169	Human Diseases	Infectious diseases: Viral
Transcriptional misregulation in cancer	338	ko05202	Human Diseases	Cancers: Overview
Amoebiasis	326	ko05146	Human Diseases	Infectious diseases: Parasitic
Alzheimer's disease	295	ko05010	Human Diseases	Neurodegenerative diseases
Vibrio cholerae infection	290	ko05110	Human Diseases	Infectious diseases: Bacterial
Influenza A	260	ko05164	Human Diseases	Infectious diseases: Viral
Herpes simplex infection	248	ko05168	Human Diseases	Infectious diseases: Viral
Dilated cardiomyopathy	230	ko05414	Human Diseases	Cardiovascular diseases
Hypertrophic cardiomyopathy (HCM)	225	ko05410	Human Diseases	Cardiovascular diseases
Tuberculosis	218	ko05152	Human Diseases	Infectious diseases: Bacterial
Salmonella infection	212	ko05132	Human Diseases	Infectious diseases: Bacterial
Parkinson's disease	208	ko05012	Human Diseases	Neurodegenerative diseases
Pathogenic Escherichia coli infection	150	ko05130	Human Diseases	Infectious diseases: Bacterial

Bacterial invasion of epithelial cells	147	ko05100	Human Diseases	Infectious diseases: Bacterial
Viral myocarditis	142	ko05416	Human Diseases	Cardiovascular diseases
Toxoplasmosis	136	ko05145	Human Diseases	Infectious diseases: Parasitic
Prostate cancer	133	ko05215	Human Diseases	Cancers: Specific types
Alcoholism	132	ko05034	Human Diseases	Substance dependence
Measles	132	ko05162	Human Diseases	Infectious diseases: Viral
Shigellosis	118	ko05131	Human Diseases	Infectious diseases: Bacterial
Amphetamine addiction	113	ko05031	Human Diseases	Substance dependence
Small cell lung cancer	113	ko05222	Human Diseases	Cancers: Specific types
Renal cell carcinoma	112	ko05211	Human Diseases	Cancers: Specific types
Amyotrophic lateral sclerosis (ALS)	110	ko05014	Human Diseases	Neurodegenerative diseases
Hepatitis C	107	ko05160	Human Diseases	Infectious diseases: Viral
Prion diseases	100	ko05020	Human Diseases	Neurodegenerative diseases
Glioma	100	ko05214	Human Diseases	Cancers: Specific types
Arrhythmogenic right ventricular cardiomyopathy (ARVC)	98	ko05412	Human Diseases	Cardiovascular diseases
Morphine addiction	96	ko05032	Human Diseases	Substance dependence
Epithelial cell signaling in Helicobacter pylori infection	94	ko05120	Human Diseases	Infectious diseases: Bacterial
Colorectal cancer	88	ko05210	Human Diseases	Cancers: Specific types
Legionellosis	88	ko05134	Human Diseases	Infectious diseases: Bacterial
Basal cell carcinoma	86	ko05217	Human Diseases	Cancers: Specific types
Endometrial cancer	81	ko05213	Human Diseases	Cancers: Specific types
Chagas disease (American trypanosomiasis)	78	ko05142	Human Diseases	Infectious diseases: Parasitic
Type II diabetes mellitus	72	ko04930	Human Diseases	Endocrine and metabolic diseases
Chronic myeloid leukemia	71	ko05220	Human Diseases	Cancers: Specific types
Thyroid cancer	62	ko05216	Human Diseases	Cancers: Specific types
Rheumatoid arthritis	61	ko05323	Human Diseases	Immune diseases
Malaria	60	ko05144	Human Diseases	Infectious diseases: Parasitic
Pertussis	60	ko05133	Human Diseases	Infectious diseases: Bacterial
Pancreatic cancer	60	ko05212	Human Diseases	Cancers: Specific types
Cocaine addiction	59	ko05030	Human Diseases	Substance dependence
Non-small cell lung cancer	55	ko05223	Human Diseases	Cancers: Specific types
Acute myeloid leukemia	55	ko05221	Human Diseases	Cancers: Specific types
Systemic lupus erythematosus	49	ko05322	Human Diseases	Immune diseases
Nicotine addiction	48	ko05033	Human Diseases	Substance dependence

Staphylococcus aureus infection	48	ko05150	Human Diseases	Infectious diseases: Bacterial
Melanoma	46	ko05218	Human Diseases	Cancers: Specific types
Leishmaniasis	45	ko05140	Human Diseases	Infectious diseases: Parasitic
Bladder cancer	41	ko05219	Human Diseases	Cancers: Specific types
Maturity onset diabetes of the young	33	ko04950	Human Diseases	Endocrine and metabolic diseases
Autoimmune thyroid disease	32	ko05320	Human Diseases	Immune diseases
Primary immunodeficiency	21	ko05340	Human Diseases	Immune diseases
African trypanosomiasis	19	ko05143	Human Diseases	Infectious diseases: Parasitic
Type I diabetes mellitus	17	ko04940	Human Diseases	Endocrine and metabolic diseases
Asthma	2	ko05310	Human Diseases	Immune diseases
Allograft rejection	1	ko05330	Human Diseases	Immune diseases
Graft-versus-host disease	1	ko05332	Human Diseases	Immune diseases
Metabolic pathways	2105	ko01100	Metabolism	Global map
Purine metabolism	390	ko00230	Metabolism	Nucleotide metabolism
Pyrimidine metabolism	301	ko00240	Metabolism	Nucleotide metabolism
Lysine degradation	213	ko00310	Metabolism	Amino acid metabolism
Oxidative phosphorylation	195	ko00190	Metabolism	Energy metabolism
Starch and sucrose metabolism	153	ko00500	Metabolism	Carbohydrate metabolism
Glycerophospholipid metabolism	139	ko00564	Metabolism	Lipid metabolism
Glycine, serine and threonine metabolism	138	ko00260	Metabolism	Amino acid metabolism
Drug metabolism - other enzymes	134	ko00983	Metabolism	Xenobiotics biodegradation and metabolism
Retinol metabolism	125	ko00830	Metabolism	Metabolism of cofactors and vitamins
Inositol phosphate metabolism	122	ko00562	Metabolism	Carbohydrate metabolism
Glycerolipid metabolism	121	ko00561	Metabolism	Lipid metabolism
Amino sugar and nucleotide sugar metabolism	115	ko00520	Metabolism	Carbohydrate metabolism
Pentose and glucuronate interconversions	115	ko00040	Metabolism	Carbohydrate metabolism
Drug metabolism - cytochrome P450	114	ko00982	Metabolism	Xenobiotics biodegradation and metabolism
Glutathione metabolism	113	ko00480	Metabolism	Metabolism of other amino acids
Metabolism of xenobiotics by cytochrome P450	112	ko00980	Metabolism	Xenobiotics biodegradation and metabolism
Other types of O-glycan biosynthesis	111	ko00514	Metabolism	Glycan biosynthesis and metabolism
Galactose metabolism	106	ko00052	Metabolism	Carbohydrate metabolism

Porphyrin and chlorophyll metabolism	103	ko00860	Metabolism	Metabolism of cofactors and vitamins
Steroid hormone biosynthesis	101	ko00140	Metabolism	Lipid metabolism
Pyruvate metabolism	94	ko00620	Metabolism	Carbohydrate metabolism
Arginine and proline metabolism	94	ko00330	Metabolism	Amino acid metabolism
Ascorbate and aldarate metabolism	92	ko00053	Metabolism	Carbohydrate metabolism
Glycolysis / Gluconeogenesis	91	ko00010	Metabolism	Carbohydrate metabolism
Tyrosine metabolism	90	ko00350	Metabolism	Amino acid metabolism
Pentose phosphate pathway	84	ko00030	Metabolism	Carbohydrate metabolism
Valine, leucine and isoleucine degradation	82	ko00280	Metabolism	Amino acid metabolism
N-Glycan biosynthesis	81	ko00510	Metabolism	Glycan biosynthesis and metabolism
Tryptophan metabolism	79	ko00380	Metabolism	Amino acid metabolism
Fructose and mannose metabolism	76	ko00051	Metabolism	Carbohydrate metabolism
Fatty acid metabolism	72	ko00071	Metabolism	Lipid metabolism
Cysteine and methionine metabolism	70	ko00270	Metabolism	Amino acid metabolism
Glycosphingolipid biosynthesis - ganglio series	60	ko00604	Metabolism	Glycan biosynthesis and metabolism
Sphingolipid metabolism	60	ko00600	Metabolism	Lipid metabolism
Butanoate metabolism	60	ko00650	Metabolism	Carbohydrate metabolism
Glycosylphosphatidylinositol(GPI)-anchor biosynthesis	59	ko00563	Metabolism	Glycan biosynthesis and metabolism
Fatty acid elongation	58	ko00062	Metabolism	Lipid metabolism
Fatty acid biosynthesis	56	ko00061	Metabolism	Lipid metabolism
beta-Alanine metabolism	54	ko00410	Metabolism	Metabolism of other amino acids
Citrate cycle (TCA cycle)	54	ko00020	Metabolism	Carbohydrate metabolism
alpha-Linolenic acid metabolism	52	ko00592	Metabolism	Lipid metabolism
Alanine, aspartate and glutamate metabolism	51	ko00250	Metabolism	Amino acid metabolism
Insect hormone biosynthesis	50	ko00981	Metabolism	Metabolism of terpenoids and polyketides
Glycosaminoglycan biosynthesis - heparan sulfate	49	ko00534	Metabolism	Glycan biosynthesis and metabolism
Propanoate metabolism	46	ko00640	Metabolism	Carbohydrate metabolism
Terpenoid backbone biosynthesis	46	ko00900	Metabolism	Metabolism of terpenoids and polyketides

Biosynthesis of unsaturated fatty acids	45	ko01040	Metabolism	Lipid metabolism
Nicotinate and nicotinamide metabolism	43	ko00760	Metabolism	Metabolism of cofactors and vitamins
Arachidonic acid metabolism	42	ko00590	Metabolism	Lipid metabolism
Phenylalanine metabolism	42	ko00360	Metabolism	Amino acid metabolism
Glyoxylate and dicarboxylate metabolism	41	ko00630	Metabolism	Carbohydrate metabolism
Other glycan degradation	39	ko00511	Metabolism	Glycan biosynthesis and metabolism
One carbon pool by folate	37	ko00670	Metabolism	Metabolism of cofactors and vitamins
Linoleic acid metabolism	32	ko00591	Metabolism	Lipid metabolism
Glycosaminoglycan degradation	32	ko00531	Metabolism	Glycan biosynthesis and metabolism
Steroid biosynthesis	32	ko00100	Metabolism	Lipid metabolism
Riboflavin metabolism	30	ko00740	Metabolism	Metabolism of cofactors and vitamins
Ether lipid metabolism	30	ko00565	Metabolism	Lipid metabolism
Histidine metabolism	29	ko00340	Metabolism	Amino acid metabolism
Glycosaminoglycan biosynthesis - chondroitin sulfate	29	ko00532	Metabolism	Glycan biosynthesis and metabolism
Folate biosynthesis	29	ko00790	Metabolism	Metabolism of cofactors and vitamins
Selenocompound metabolism	25	ko00450	Metabolism	Metabolism of other amino acids
Cyanoamino acid metabolism	25	ko00460	Metabolism	Metabolism of other amino acids
Taurine and hypotaurine metabolism	22	ko00430	Metabolism	Metabolism of other amino acids
Pantothenate and CoA biosynthesis	20	ko00770	Metabolism	Metabolism of cofactors and vitamins
Glycosaminoglycan biosynthesis - keratan sulfate	18	ko00533	Metabolism	Glycan biosynthesis and metabolism
Synthesis and degradation of ketone bodies	17	ko00072	Metabolism	Lipid metabolism
Glycosphingolipid biosynthesis - globo series	15	ko00603	Metabolism	Glycan biosynthesis and metabolism
Mucin type O-Glycan biosynthesis	15	ko00512	Metabolism	Glycan biosynthesis and metabolism
Ubiquinone and other terpenoid-quinone biosynthesis	14	ko00130	Metabolism	Metabolism of cofactors and vitamins
Sulfur metabolism	13	ko00920	Metabolism	Energy metabolism

Glycosphingolipid biosynthesis - lacto and neolacto series	13	ko00601	Metabolism	Glycan biosynthesis and metabolism
Valine, leucine and isoleucine biosynthesis	12	ko00290	Metabolism	Amino acid metabolism
Primary bile acid biosynthesis	11	ko00120	Metabolism	Lipid metabolism
Vitamin B6 metabolism	8	ko00750	Metabolism	Metabolism of cofactors and vitamins
Butirosin and neomycin biosynthesis	8	ko00524	Metabolism	Biosynthesis of other secondary metabolites
Lipoic acid metabolism	7	ko00785	Metabolism	Metabolism of cofactors and vitamins
Phenylalanine, tyrosine and tryptophan biosynthesis	7	ko00400	Metabolism	Amino acid metabolism
Biotin metabolism	7	ko00780	Metabolism	Metabolism of cofactors and vitamins
D-Arginine and D-ornithine metabolism	6	ko00472	Metabolism	Metabolism of other amino acids
Thiamine metabolism	5	ko00730	Metabolism	Metabolism of cofactors and vitamins
Lysine biosynthesis	4	ko00300	Metabolism	Amino acid metabolism
Caffeine metabolism	4	ko00232	Metabolism	Biosynthesis of other secondary metabolites
D-Glutamine and D-glutamate metabolism	1	ko00471	Metabolism	Metabolism of other amino acids
Vascular smooth muscle contraction	398	ko04270	Organismal Systems	Circulatory system
Bile secretion	274	ko04976	Organismal Systems	Digestive system
Insulin signaling pathway	270	ko04910	Organismal Systems	Endocrine system
Pancreatic secretion	225	ko04972	Organismal Systems	Digestive system
Salivary secretion	205	ko04970	Organismal Systems	Digestive system
Neurotrophin signaling pathway	199	ko04722	Organismal Systems	Nervous system
Chemokine signaling pathway	193	ko04062	Organismal Systems	Immune system
Dopaminergic synapse	189	ko04728	Organismal Systems	Nervous system
Protein digestion and absorption	186	ko04974	Organismal Systems	Digestive system
Axon guidance	182	ko04360	Organismal Systems	Development
Leukocyte transendothelial migration	180	ko04670	Organismal Systems	Immune system
Gastric acid secretion	178	ko04971	Organismal Systems	Digestive system
Dorso-ventral axis formation	169	ko04320	Organismal Systems	Development

Cardiac muscle contraction	154	ko04260	Organismal Systems	Circulatory system
Melanogenesis	146	ko04916	Organismal Systems	Endocrine system
Fc gamma R-mediated phagocytosis	146	ko04666	Organismal Systems	Immune system
Long-term potentiation	143	ko04720	Organismal Systems	Nervous system
Progesterone-mediated oocyte maturation	140	ko04914	Organismal Systems	Endocrine system
GnRH signaling pathway	137	ko04912	Organismal Systems	Endocrine system
Glutamatergic synapse	132	ko04724	Organismal Systems	Nervous system
Vitamin digestion and absorption	130	ko04977	Organismal Systems	Digestive system
Synaptic vesicle cycle	129	ko04721	Organismal Systems	Nervous system
T cell receptor signaling pathway	122	ko04660	Organismal Systems	Immune system
Cholinergic synapse	119	ko04725	Organismal Systems	Nervous system
PPAR signaling pathway	105	ko03320	Organismal Systems	Endocrine system
Phototransduction - fly	104	ko04745	Organismal Systems	Sensory system
Adipocytokine signaling pathway	102	ko04920	Organismal Systems	Endocrine system
Antigen processing and presentation	102	ko04612	Organismal Systems	Immune system
Retrograde endocannabinoid signaling	100	ko04723	Organismal Systems	Nervous system
Hematopoietic cell lineage	97	ko04640	Organismal Systems	Immune system
Serotonergic synapse	97	ko04726	Organismal Systems	Nervous system
Fat digestion and absorption	95	ko04975	Organismal Systems	Digestive system
Fc epsilon RI signaling pathway	91	ko04664	Organismal Systems	Immune system
GABAergic synapse	91	ko04727	Organismal Systems	Nervous system
Mineral absorption	88	ko04978	Organismal Systems	Digestive system
Vasopressin-regulated water reabsorption	86	ko04962	Organismal Systems	Excretory system
Olfactory transduction	80	ko04740	Organismal Systems	Sensory system
Complement and coagulation cascades	76	ko04610	Organismal Systems	Immune system
Cytosolic DNA-sensing pathway	75	ko04623	Organismal Systems	Immune system
Toll-like receptor signaling pathway	74	ko04620	Organismal Systems	Immune system
Osteoclast differentiation	68	ko04380	Organismal Systems	Development
Long-term depression	65	ko04730	Organismal Systems	Nervous system
Circadian rhythm - mammal	63	ko04710	Organismal Systems	Environmental adaptation
B cell receptor signaling pathway	62	ko04662	Organismal Systems	Immune system
Endocrine and other factor-regulated calcium reabsorption	60	ko04961	Organismal Systems	Excretory system
NOD-like receptor signaling pathway	60	ko04621	Organismal Systems	Immune system
Natural killer cell mediated cytotoxicity	56	ko04650	Organismal Systems	Immune system
Circadian rhythm - fly	53	ko04711	Organismal Systems	Environmental adaptation

Phototransduction	51	ko04744	Organismal Systems	Sensory system
Carbohydrate digestion and absorption	50	ko04973	Organismal Systems	Digestive system
RIG-I-like receptor signaling pathway	46	ko04622	Organismal Systems	Immune system
Renin-angiotensin system	46	ko04614	Organismal Systems	Endocrine system
Collecting duct acid secretion	44	ko04966	Organismal Systems	Excretory system
Aldosterone-regulated sodium reabsorption	33	ko04960	Organismal Systems	Excretory system
Taste transduction	28	ko04742	Organismal Systems	Sensory system
Proximal tubule bicarbonate reclamation	25	ko04964	Organismal Systems	Excretory system
